# Supplementary figures and images for: Combinations of low-level and high-level neural processes account for distinct patterns of context-dependent choice
Source: PLoS Comput Biol. 2019 Oct 14;15(10):e1007427. doi: 10.1371/journal.pcbi.1007427 (PMC6812848; doi:10.1371/journal.pcbi.1007427)

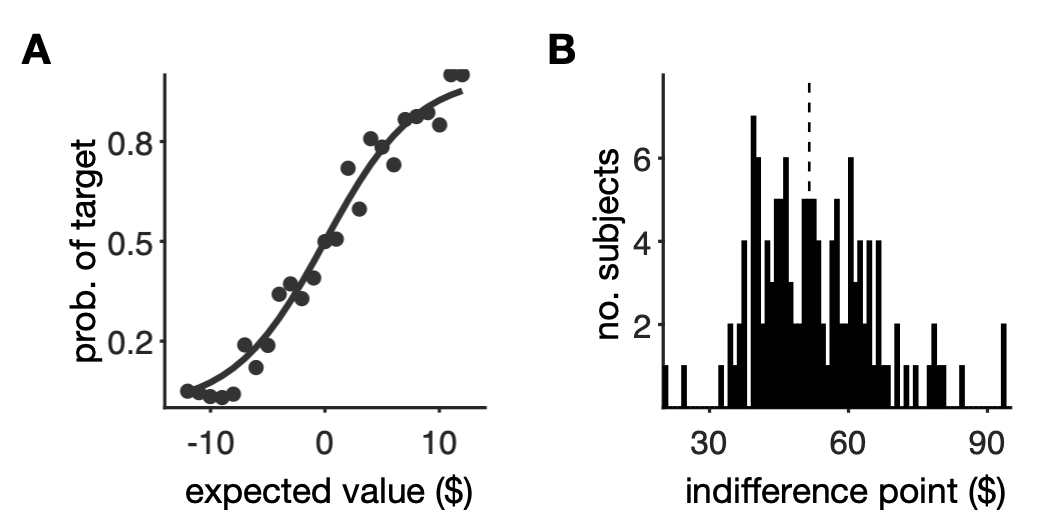

Supplement: S1 Fig — (A) Plotted is the probability of choosing the low-risk gamble (target) as a function of the reward magnitude of the high-risk gambles for an individual subject. The solid curve shows the fit using a sigmoid function. Each black dot represents an individual subject’s data. (B) Distribution of estimated indifference points in estimation task across subjects. The indifference point is defined as the magnitude of a high-risk gamble that was as equally preferred as the low-risk gamble. The dashed line indicates the median. Overall, we observed large variability for the indifference point across subjects. (TIF) [file pcbi.1007427.s001.tif]

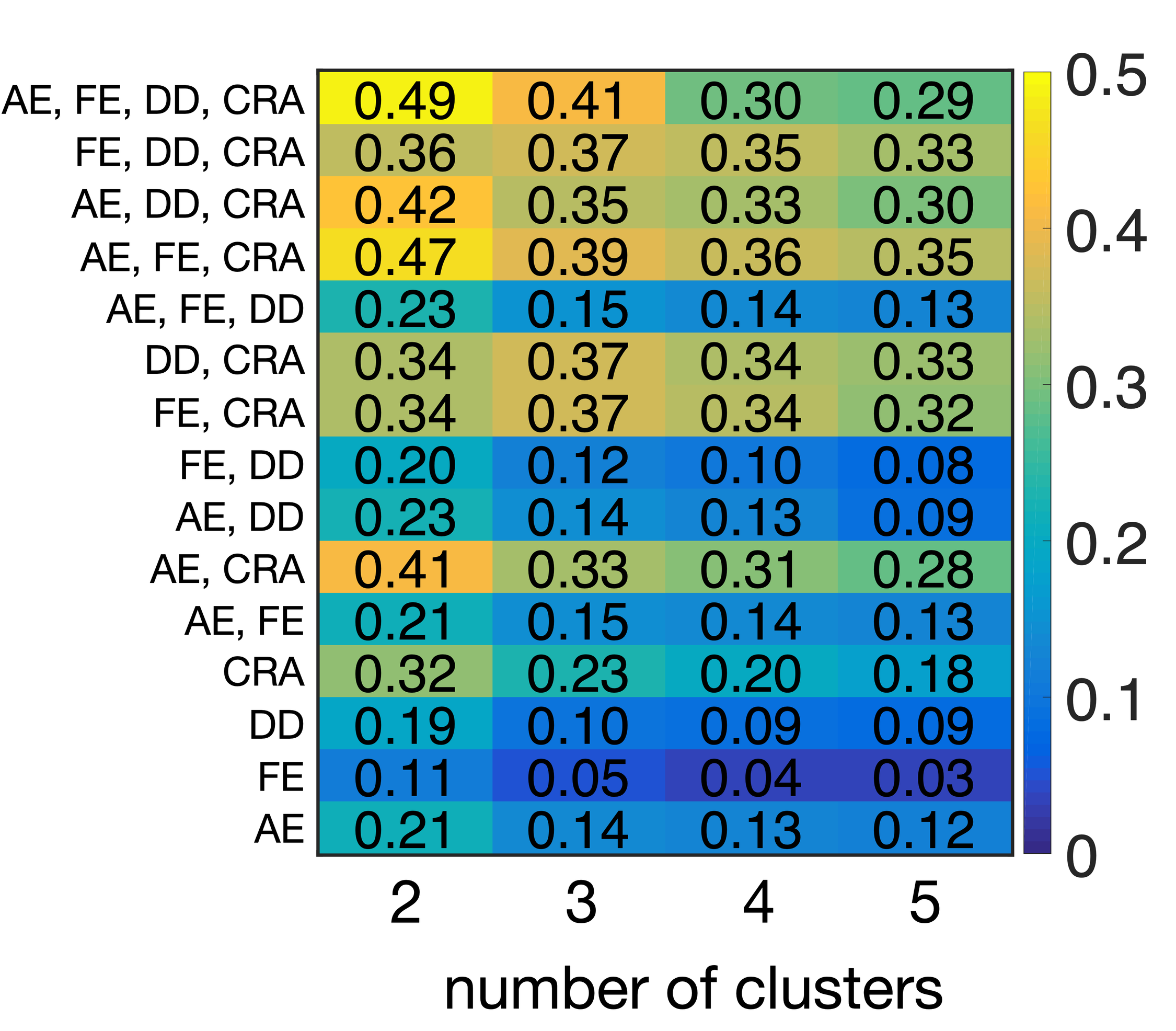

Supplement: S2 Fig — Reported are silhouette values for a given number of clusters and combination of decoy-effect indices (AE: attraction effect; FE: frequency effect; DD: dominant vs. dominated; CRA: change in risk aversion). The silhouette can take any values between -1 and 1; higher values indicate that each data point (a measure or set of measures) is closely matched to its own cluster and poorly matched to neighboring clusters. Best clustering results are achieved using all decoy-effect indices and two clusters. (TIF) [file pcbi.1007427.s002.tif]

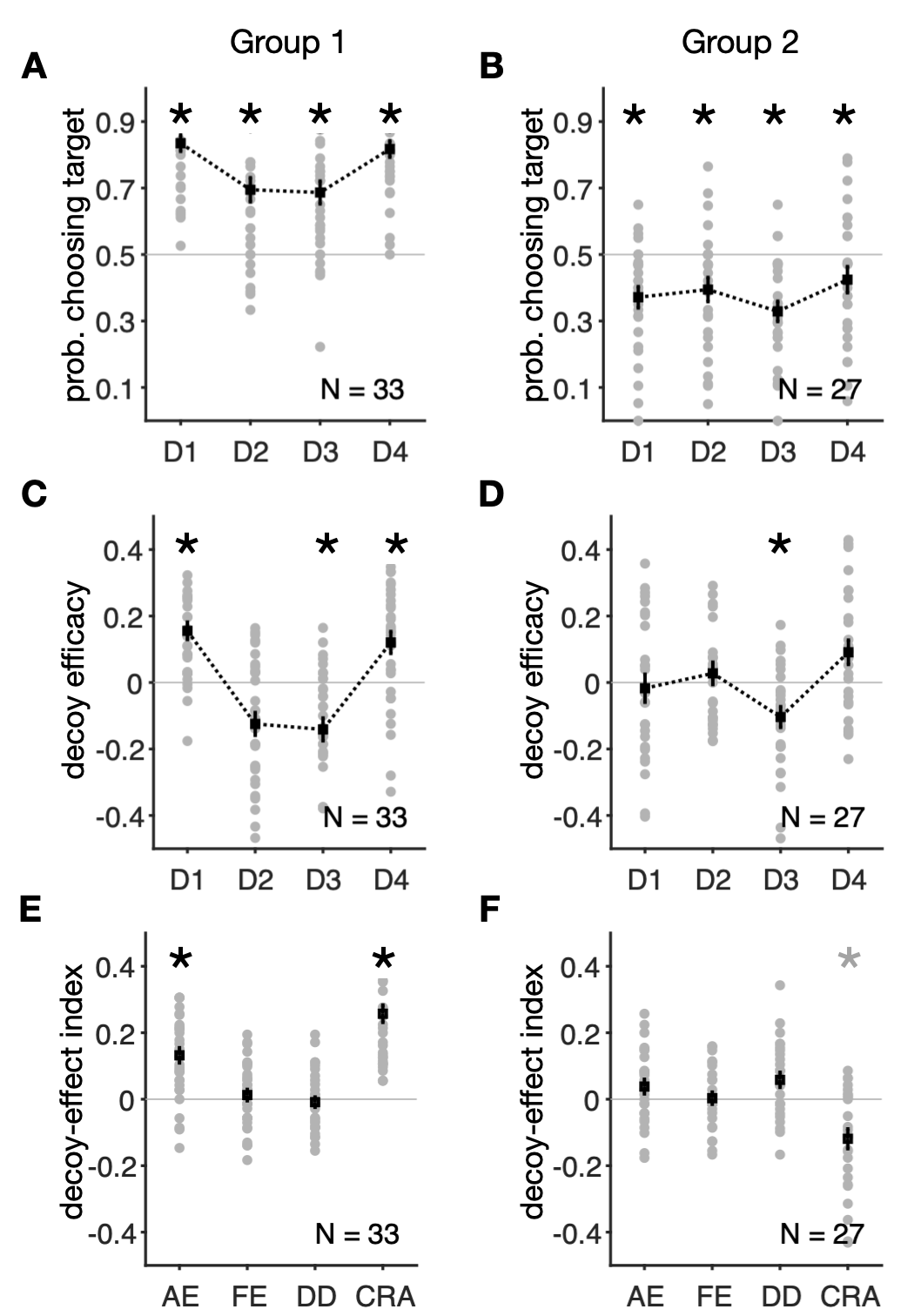

Supplement: S3 Fig — (A–B) Probability of selecting the target for different decoy types in the two groups of subjects identified by clustering. Each gray circle shows the average probability that an individual subject selected the target for a given decoy location, and black squares indicate the average probability across all subjects. Error bars show the s.e.m., and an asterisk shows that the median of choice probability across subjects for a given decoy location is significantly different from 0.5 (two-sided Wilcoxon signed-test, p < 0.05). A gray asterisk indicates that the difference is not significant after Bonferroni correction. (C–D) Decoy efficacies in the two groups of subjects. Subjects in Group 1 exhibited strong, consistent decoy effects (C), whereas the decoy effects were inconsistent in Group 2 (D). (E–F) Plot shows decoy-effect indices for individuals in the two groups of subjects. The first group showed a strong attraction effect and an overall increase in risk aversion. The second group showed a significant decrease in risk aversion. (TIF) [file pcbi.1007427.s003.tif]

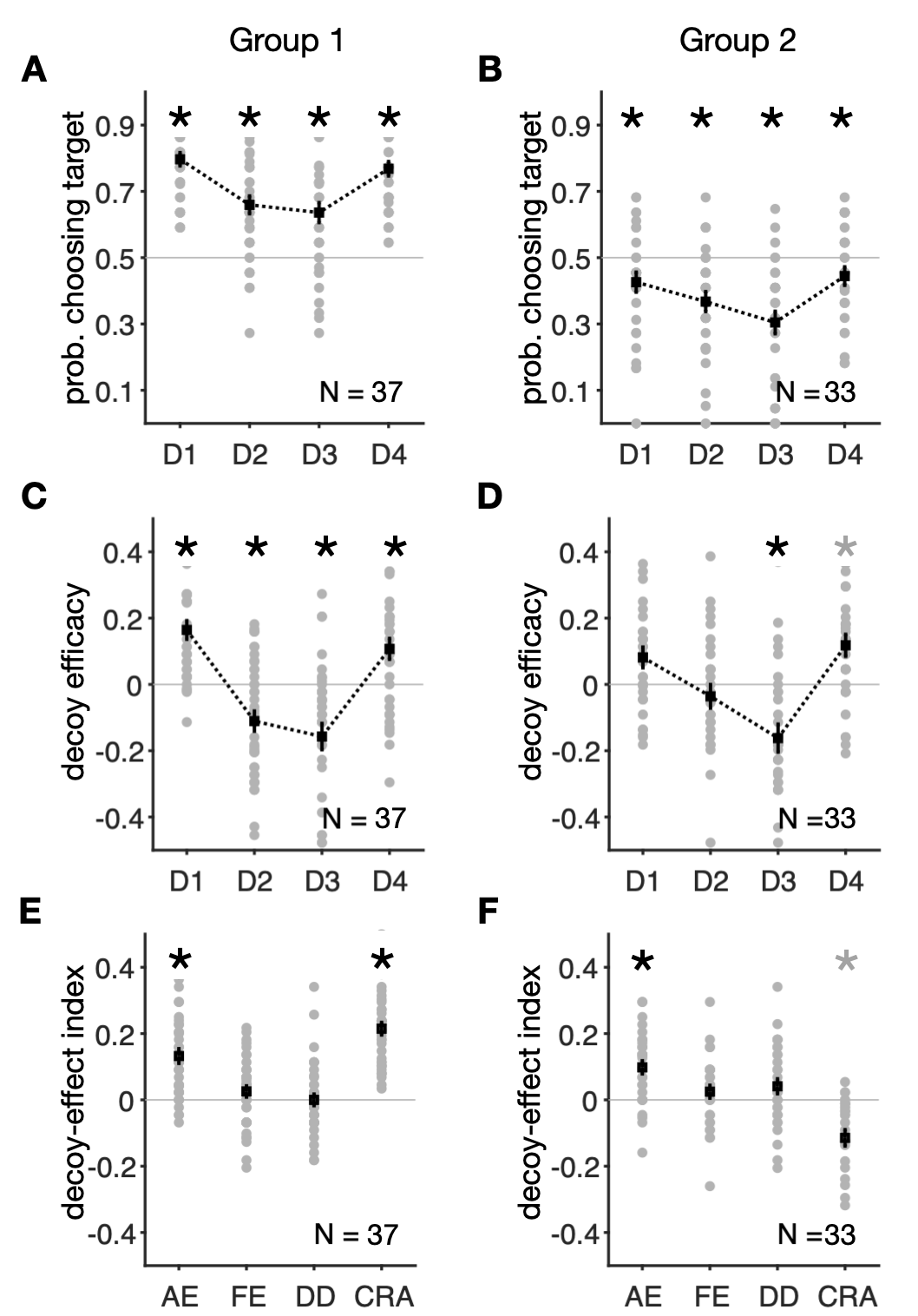

Supplement: S4 Fig — (A–B) Probability of selecting the target for different decoy types in the two groups of subjects identified by clustering. Each gray circle shows the average probability that an individual subject selected the target for a given decoy location, and black squares indicate the average probability across all subjects. Error bars show the s.e.m., and an asterisk shows that the median of choice probability across subjects for a given decoy location is significantly different from 0.5 (two-sided Wilcoxon signed-test, p < 0.05). A gray asterisk indicates that the difference is not significant after Bonferroni correction. (C–D) Decoy efficacies in the two groups of subjects. Subjects in Group 1 exhibited strong, consistent decoy effects (C), whereas the decoy effects were inconsistent and limited to decoys next to the less risky gamble (target) in Group 2 (D). (E–F) Plot shows decoy-effect indices for individuals in the two groups of subjects. The first group showed a strong attraction effect and an overall increase in risk aversion. The second group showed a significant attraction effect and a decrease in risk aversion. (TIF) [file pcbi.1007427.s004.tif]

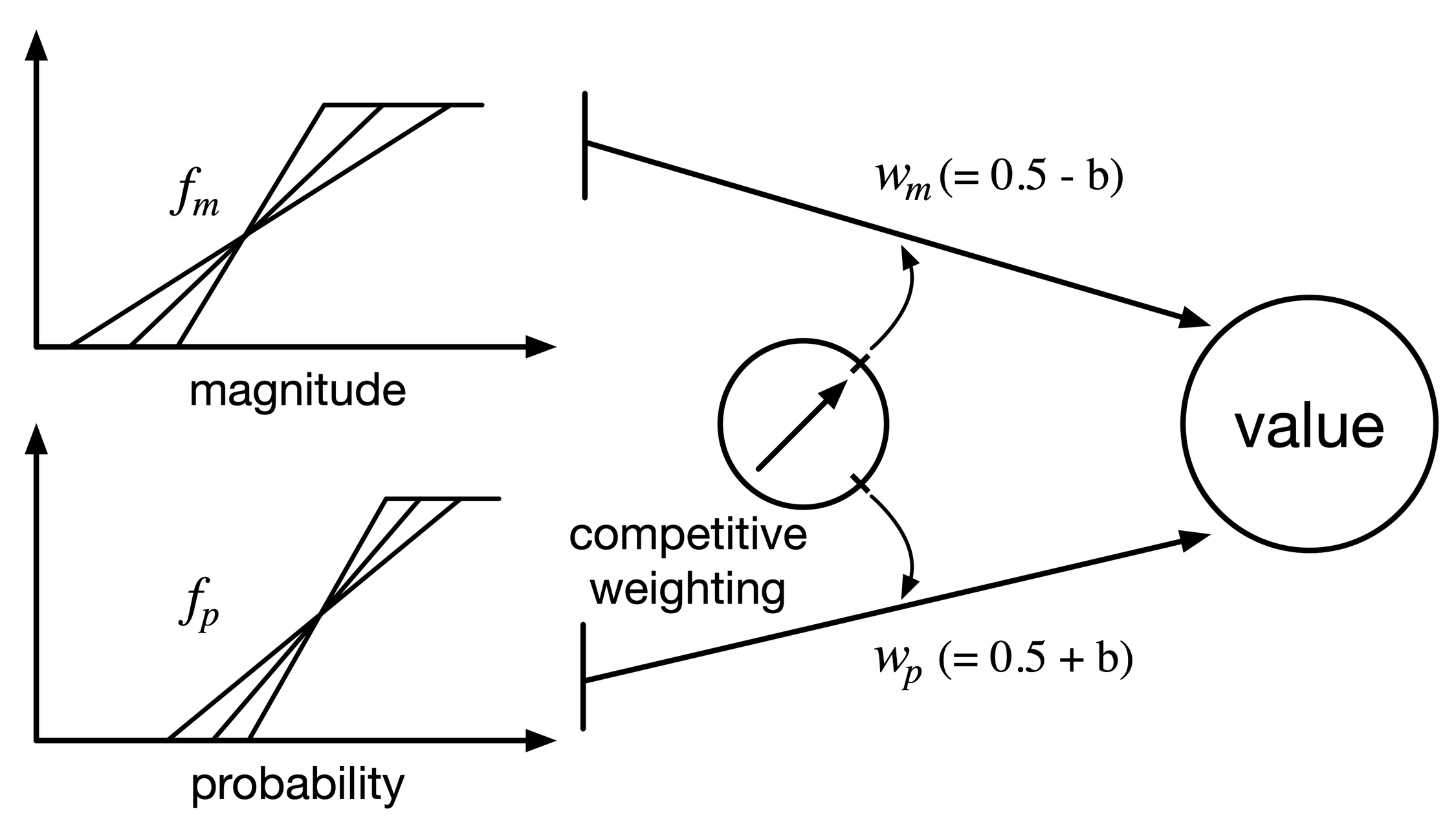

Supplement: S5 Fig — Values of gambles in a given attribute (reward magnitude and probability) are encoded by the corresponding attribute-encoding population of neurons. We assumed the response of attribute-encoding neurons (response curve) to be a linear function of the attribute value. Representation factor fi (i = {p,m}) determines dynamic range (threshold and saturation points) of neural response as well as the slope of the response curve for each attribute. We assumed that an additional competitive mechanism could modulate the weight of each attribute (wm and wp) on the overall value of each gamble. (TIF) [file pcbi.1007427.s005.tif]

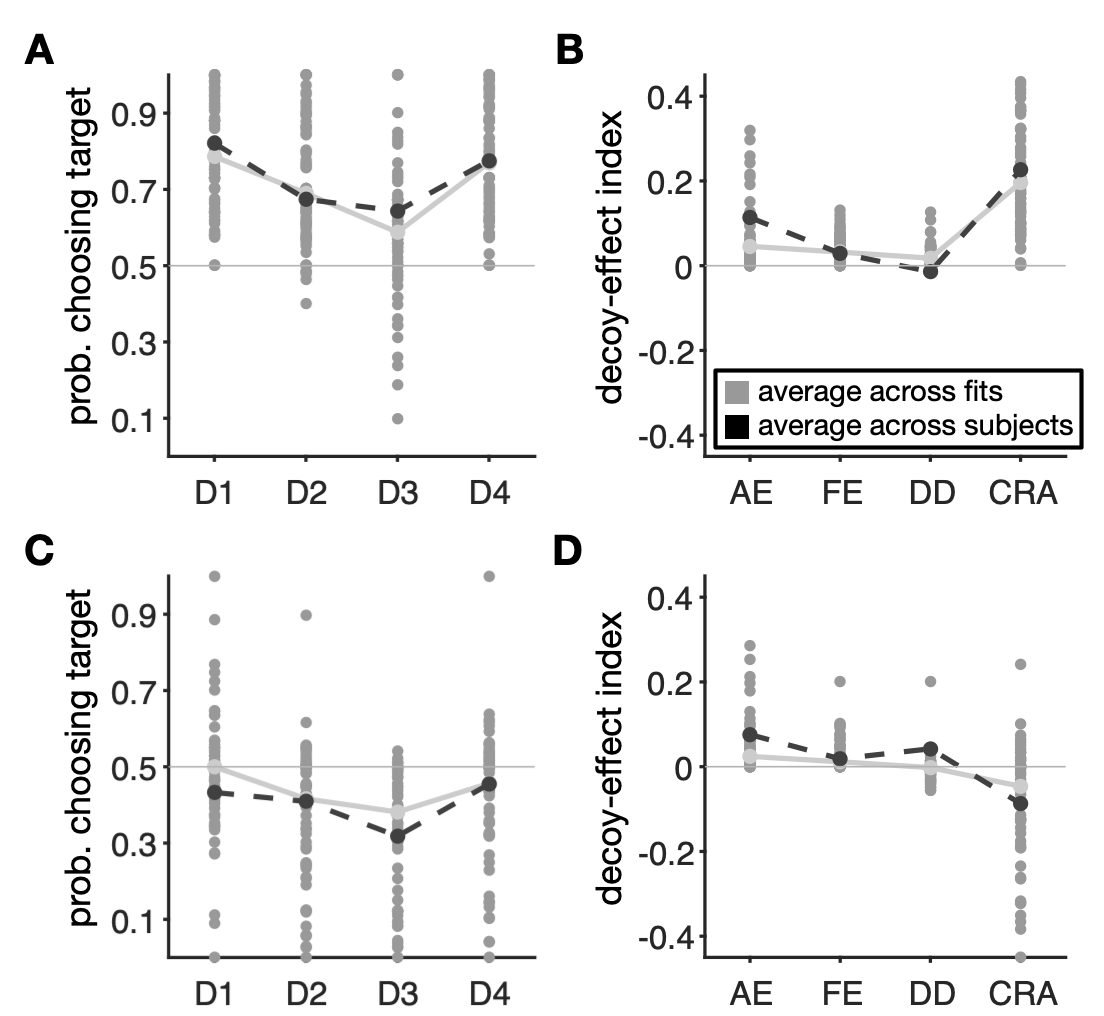

Supplement: S6 Fig — Plotted are fit of individual subjects based on a model that has adjustments to decoy presentation similar to the original model by Soltani et al. [23]. (A, C) Each gray circle shows the probability of selecting the target for different decoy types based on the model fit for individual subjects in the first (A) and second (C) groups of subjects. For comparison, the average value across subjects (black dashed lines) and their fits (gray solid lines) are plotted as well. (B, D) Plots show four measures for quantifying different effects of decoys on preference based on the cited model fit for individual subjects in the first (B) and second (D) groups of subjects (AE: attraction effect; FE: frequency effect; DD: dominant vs. dominated; and CRA: change in risk aversion). (TIF) [file pcbi.1007427.s006.tif]

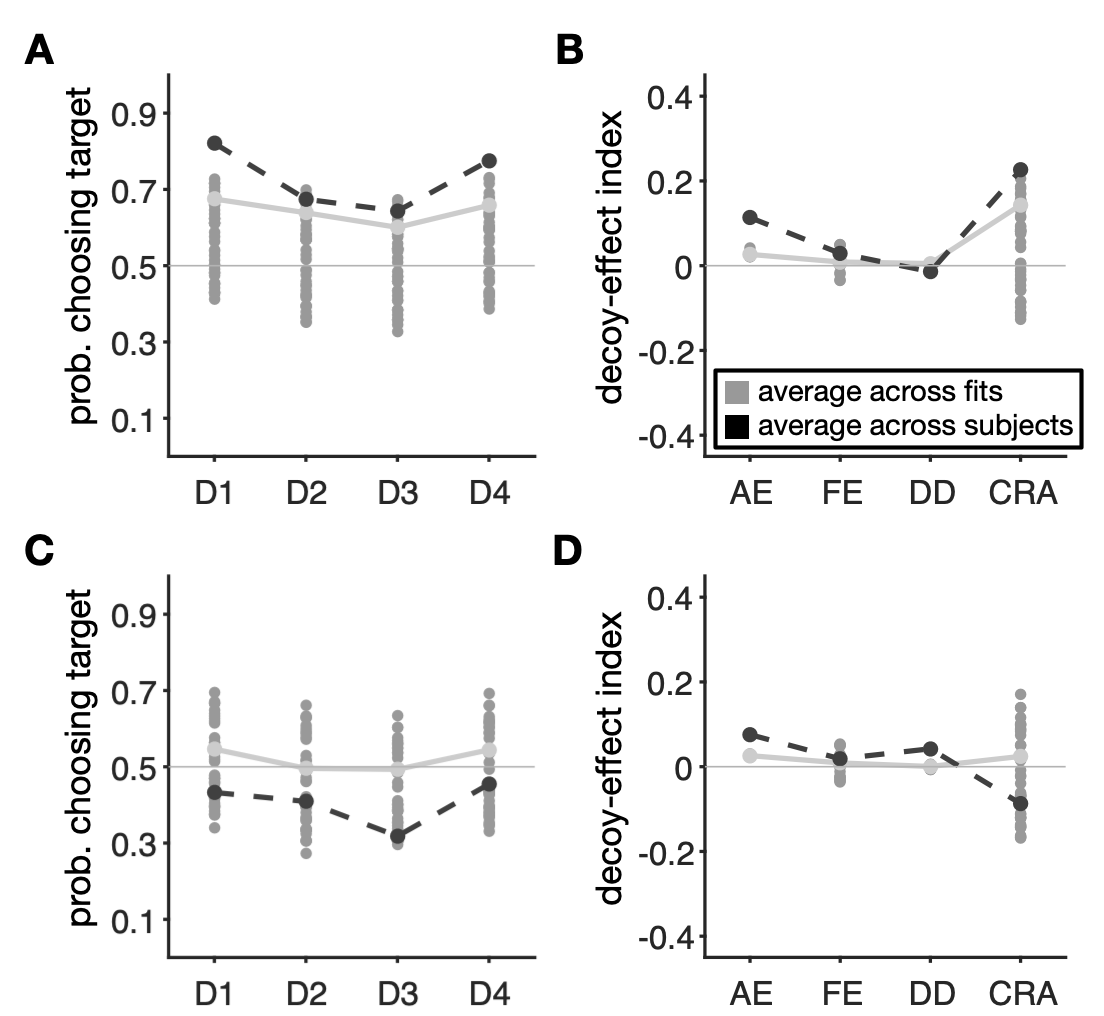

Supplement: S7 Fig — Plotted are fit of individual subjects based on a model that only assumes a competitive mechanism to modulate the weight of each attribute (wm and wp) on the overall value of each gamble. (A, C) Each gray circle shows the probability of selecting the target for different decoy types based on the model fit for individual subjects in the first (A) and second (C) groups of subjects. For comparison, the average value across subjects (black dashed line) and their fits (gray solid lines) are plotted as well. (B, D) Plots show four measures for quantifying different effects of decoys on preference based on the cited model fit for individual subjects in the first (B) and second (D) groups of subjects (AE: attraction effect; FE: frequency effect; DD: dominant vs. dominated; and CRA: change in risk aversion). (TIF) [file pcbi.1007427.s007.tif]

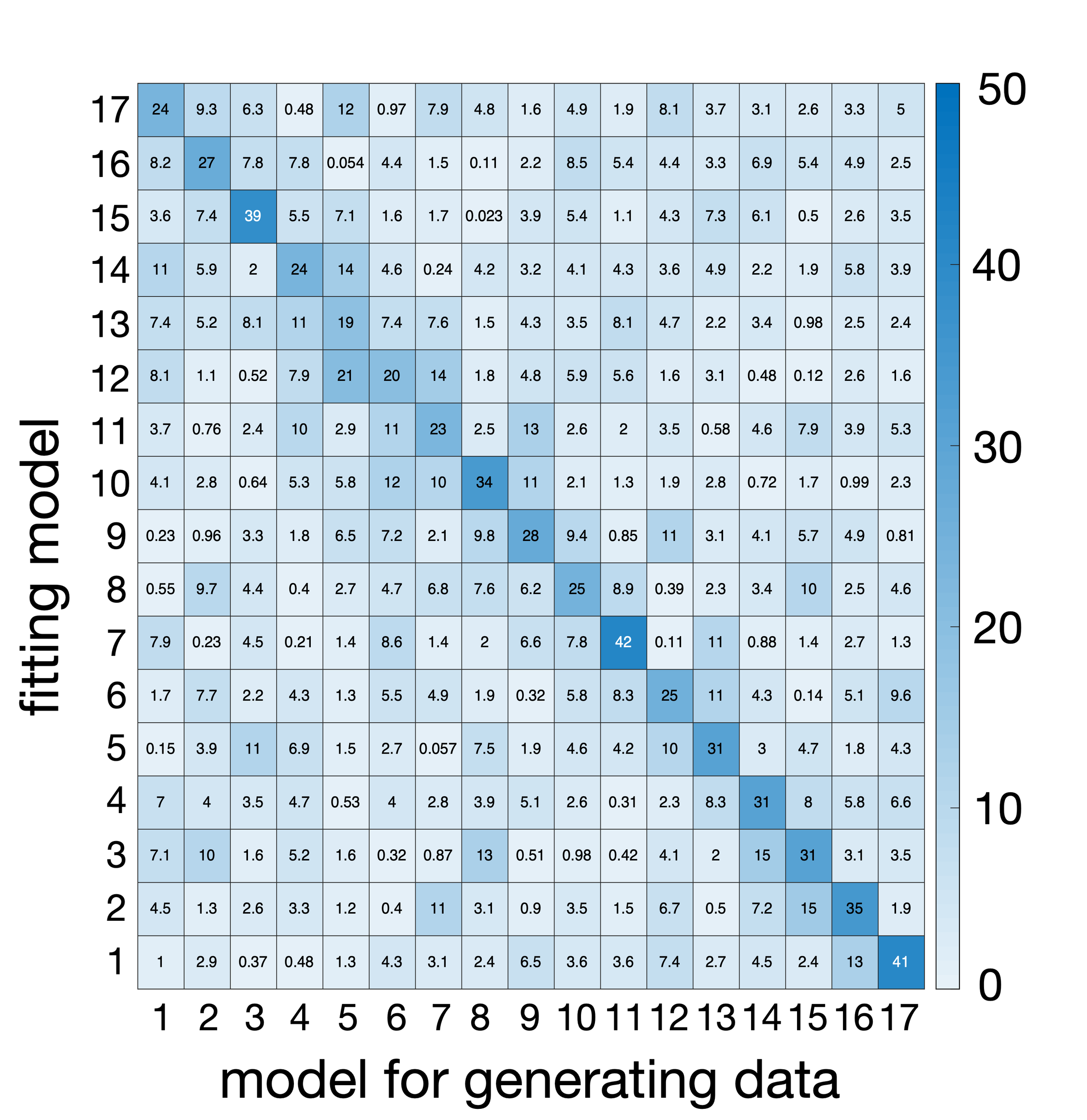

Supplement: S8 Fig — Our fitting method was able to identify the correct model. The value of each cell reports the percentage of instances that a model used to generate the data (shown on the x-axis) was best fit by the same or other models (fitting model, shown on the y-axis). The model corresponding to each number is provided in Table 1. The model with the minimum AIC was assigned as the best model. The probability for assigning the best model by chance is ~6% and thus, values above 19% on the diagonal indicate that in most cases the correct model was identified. For these simulations, we generated 200 sets of data based on a given model using parameters from the fit of individual subjects’ data. We then fit those data with all the models in order to calculate AIC and determine the best fitting model. (TIF) [file pcbi.1007427.s008.tif]

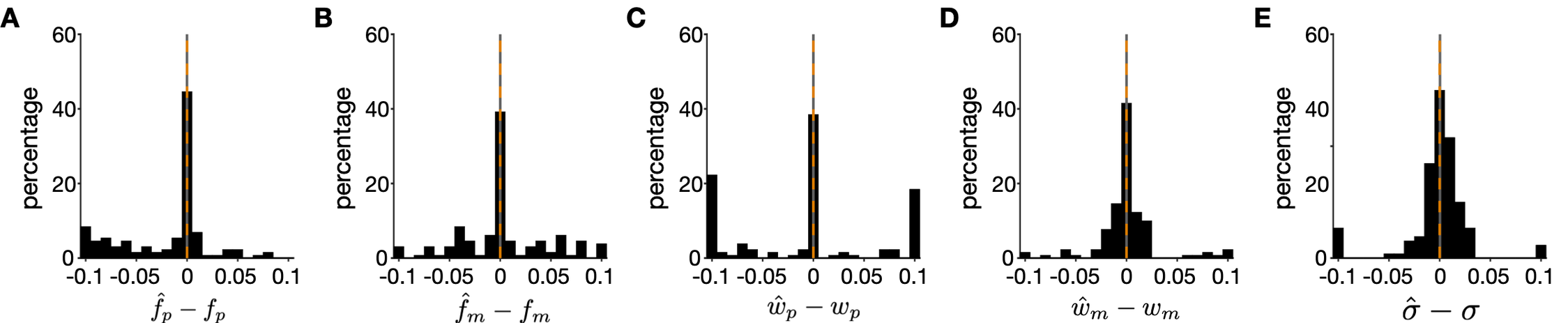

Supplement: S9 Fig — Our method was able to provide unbiased estimates of 5 model parameters. Plots show the difference between estimated and real values for fp (A), fm (B), wp (C), wm (D), and σ (E). Orange dashed and solid gray lines show zero and median, respectively. None of the differences are significantly different from zero. Estimation errors are calculated based on 500 sets of randomly generated model parameters. (TIF) [file pcbi.1007427.s009.tif]

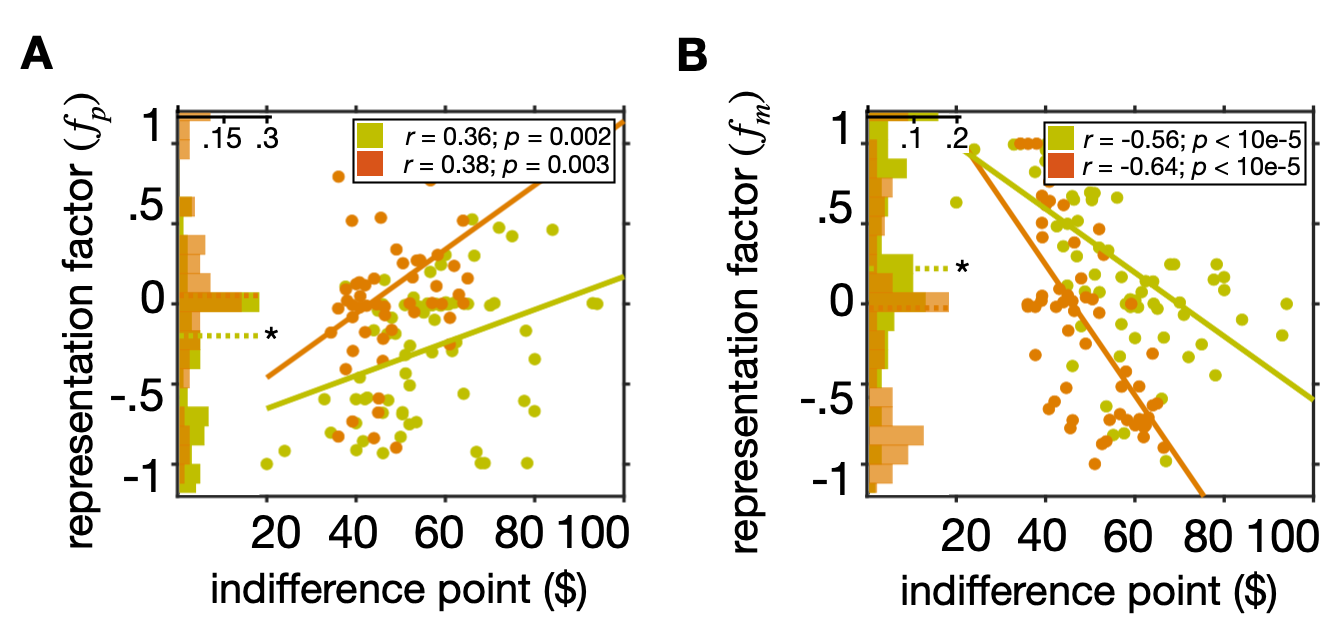

Supplement: S10 Fig — Plots show the estimated representation factor for probability (A) and magnitude (B) as a function of the indifference points within each individual. The green and orange inset histograms plot the fractions of subjects (green: Group 1; orange: Group 2) with certain values of representation factor. Neural representations to decoy presentation of reward attributes were both strongly correlated with the original degree of risk aversion in both groups, reflecting competitive adjustments in how the two reward attributes were processed in the decoy task. (TIF) [file pcbi.1007427.s010.tif]
