# Supplementary material for: Combinations of low-level and high-level neural processes account for distinct patterns of context-dependent choice
Source: PLoS Comput Biol. 2019 Oct 14;15(10):e1007427. doi: 10.1371/journal.pcbi.1007427 (PMC6812848; doi:10.1371/journal.pcbi.1007427)
Supplement: S1 Table — Reported are p-values for comparison of a pair of decoy types (rows) and for different quantities and experimental conditions (columns). The orange shading indicates p-values that are smaller than 0.05 and thus differences that are statistically significant. (DOCX) [file pcbi.1007427.s011.docx]

| comparison | prob. choosing target  (control) | decoy efficacy  (control) | prob. choosing target  (range-manipulation) | decoy efficacy  (range-manipulation) |
| --- | --- | --- | --- | --- |
| D_1_-D_2_ | 0.0767 | 0.0001 | 0.0045 | 0.0001 |
| D_1_-D_3_ | 0.0017 | 0.0001 | 0.0230 | 0.0001 |
| D_1_-D_4_ | 0.0934 | 0.0495 | 0.0902 | 0.0437 |
| D_2_-D_3_ | 0.0142 | 0.0278 | 0.0574 | 0.0301 |
| D_2_-D_4_ | 0.0421 | 0.0001 | 0.0124 | 0.0001 |
| D_3_-D_4_ | 0.0044 | 0.0001 | 0.0539 | 0.0001 |
