# Supplementary material for: Combinations of low-level and high-level neural processes account for distinct patterns of context-dependent choice
Source: PLoS Comput Biol. 2019 Oct 14;15(10):e1007427. doi: 10.1371/journal.pcbi.1007427 (PMC6812848; doi:10.1371/journal.pcbi.1007427)
Supplement: S2 Table — Reported are p-values for comparison of a pair of decoy types (rows) and for different groups and quantities (columns). The orange shading indicates p-values that are smaller than 0.05 and thus differences that are statistically significant. (DOCX) [file pcbi.1007427.s012.docx]

| comparison | prob. choosing target  (Group 1) | decoy efficacy  (Group 1) | prob. choosing target  (Group 2) | decoy efficacy  (Group 2) |
| --- | --- | --- | --- | --- |
| D_1_-D_2_ | 0.0462 | 0.0017 | 0.0014 | 0.0001 |
| D_1_-D_3_ | 0.0033 | 0.0021 | 0.0082 | 0.0012 |
| D_1_-D_4_ | 0.0246 | 0.0356 | 0.0310 | 0.0477 |
| D_2_-D_3_ | 0.0346 | 0.0257 | 0.0307 | 0.0288 |
| D_2_-D_4_ | 0.0371 | 0.0001 | 0.0023 | 0.0001 |
| D_3_-D_4_ | 0.0096 | 0.0053 | 0.0449 | 0.0015 |
